# A Highly Sensitive SERS Technique Based on Au NPs Monolayer Film Combined with Multivariate Statistical Algorithms for Auxiliary Screening of Postmenopausal Osteoporosis

**DOI:** 10.3390/bios15090568

**Published:** 2025-08-30

**Authors:** Yun Yu, Jinlian Hu, Qidan Shen, Huifeng Xu, Shanshan Wang, Xiaoning Wang, Yuhuan Zhong, Tingting He, Hao Huang, Quanxing Hong, Erdan Huang, Xihai Li

**Affiliations:** 1 Academy of Integrative Medicine, Fujian University of Traditional Chinese Medicine, Fuzhou 350122, China; jlianei99@163.com (J.H.); shenqidan1234567@126.com (Q.S.); xuhf@fjtcm.edu.cn (H.X.); 2020044@fjtcm.edu.cn (S.W.); 2007020@fjtcm.edu.cn (X.W.); yuhuan_zhong@126.com (Y.Z.); c2019078@fjtcm.edu.cn (T.H.); 1990021@fjtcm.edu.cn (H.H.); 2003011@fjtcm.edu.cn (Q.H.); 2 College of Integrative Medicine, Fujian University of Traditional Chinese Medicine, Fuzhou 350122, China; 3 Fujian Key Laboratory of Integrative Medicine on Geriatrics, Fuzhou 350122, China; 4 Fuzhou Second General Hospital, Fuzhou 350007, China; erdhuang@163.com

**Keywords:** surface-enhanced Raman scattering, Au nanoparticles, postmenopausal osteoporosis, auxiliary screening

## Abstract

Postmenopausal osteoporosis (PMOP) has become an important public health issue. The diagnosis of PMOP relies on clinical symptoms and radiology. However, most patients with PMOP do not exhibit obvious symptoms in the early stages of this disease. This study aimed to explore the feasibility of surface-enhanced Raman scattering (SERS) technology in the auxiliary screening of PMOP. PMOP rats were induced by ovariectomy (OVX) surgery, with a Sham group and an icariin (ICA) treatment group serving as controls. A monolayer film of Au nanoparticles (NPs) was prepared using the Marangoni effect in an oil/water/oil three-phase system, and was used to detect serum SERS signals in the Sham, OVX, and ICA treatment groups. Then, the spectral diagnostic model for PMOP screening was established utilizing partial least squares (PLS) and support vector machine (SVM) algorithms. Histopathology confirmed the establishment of the PMOP rat model. The assignment of Raman peaks and the analysis of spectral differences revealed the biochemical changes associated with PMOP, including the upregulation of tyrosine levels and the downregulation of arginine, tryptophan, lipids, and collagen. When employing the PLS-SVM algorithm to simultaneously classify and discriminate three groups of samples, the diagnostic sensitivity for PMOP is 93.33%, the specificity is 96.67%, and the accuracy of three-class classification is 91.11%. This study demonstrated the potential of SERS for the auxiliary screening of PMOP.

## 1. Introduction

Postmenopausal osteoporosis (PMOP) is a systemic metabolic bone disease characterized by an imbalance between bone resorption and formation, caused by the decline in ovarian function and decreasing levels of estrogen in postmenopausal women [1,2,3]. Due to population aging, the prevalence of PMOP is rising rapidly, becoming a challenging public health issue. In the early stages of PMOP, there are no obvious clinical symptoms. As a result, most PMOP patients can only be diagnosed when they suffer from fragility fractures caused by bone loss, destruction of bone microstructure, and a decline in bone mechanical properties, leading to patient suffering, high medical expenses, and a heavy burden on family [4,5]. Therefore, enhancing early screening for PMOP and identifying high-risk populations, in order to prevent or treat PMOP, is an urgent medical issue that needs to be addressed in an aging society.

The clinical diagnosis of PMOP is based on thoracic and lumbar vertebral X-rays (e.g., computed tomography) and bone density testing (e.g., dual energy X-ray absorptiometry (DXA)) [6,7]. Currently, DXA has been considered the gold standard for the diagnosis of PMOP [8]. Moreover, laboratory tests for PMOP mainly involve the detection of bone turnover biomarkers, which are classified into bone formation biomarkers (reflecting osteoblast activity and bone formation status) and bone resorption biomarkers (reflecting osteoclast activity and bone resorption levels). For example, some studies have shown that serum procollagen type I N-terminal propeptide (PINP) and serum C-terminal telopeptides of type I collagen (CTX) can serve as sensitive biomarkers for bone formation and bone resorption, respectively [9,10]. However, there are still some issues in PMOP diagnosis. Assays for serum biomarkers of bone turnover are not reliable enough to be used alone in PMOP diagnosis. For instance, bone turnover markers could not predict the fracture risk in type 2 diabetes [11]. Additionally, in the early stages of PMOP, it is difficult to detect the level of bone loss using X-ray. Furthermore, due to its radioactivity, conducting widespread mass screening for PMOP using X-rays is challenging, and X-ray-based imaging diagnosis can only be utilized as a diagnostic tool when there is a strong clinical suspicion of PMOP. Hence, it is necessary to explore a convenient and accurate auxiliary diagnostic method for PMOP.

Currently, Raman spectroscopy, as a non-destructive method that provides molecular fingerprint information, has been widely applied in fields such as biomedicine and biochemical analysis [12,13,14]. Monzem et al. performed Raman spectroscopy in mouse tibia in vivo to analyze the differences between osteoporotic bone and an healthy bone [15]. Beattie et al. employed Raman technology to act as the predictive tool for monitoring pharmacological therapy of PMOP in ovariectomised rats. In this work, micro-CT was used to evaluate tissue morphology changes, while Raman spectroscopy was employed to analyze biochemical changes in bone collagen and minerals [16]. Recently, this research team further utilized Raman spectroscopy to analyze the biochemical changes in keratin structure of PMOP patients before and after the intervention with bone active medication [17]. Chen et al. measured Raman spectra of femoral heads from patients with hip fractures and then aligned the Raman spectral information with bone density to predict osteoporosis [18]. Above studies have verified the capacity of Raman spectroscopy to analyze the biochemical composition of bone. However, the Raman scattering cross-section of molecules is exceedingly small, leading to an extremely inefficient of Raman scattering [19,20]. Therefore, the strong autofluorescence interference present a significant challenge for Raman measurements of biological samples.

Fortunately, by adsorption of sample molecules to Au or Ag nano-structures, the Raman scattering signal can be enhanced by several orders of magnitude, owing to the localized surface plasmon resonance, and this phenomenon is known as surface-enhanced Raman scattering (SERS) [21,22,23]. Due to the ultra-high sensitivity of SERS, there is a growing interest in the potential applications of SERS technology in molecular diagnostics [24,25,26,27]. Recently, SERS has received widespread attention for its application in detecting osteogenic differentiation of bone marrow mesenchymal stem cells (BMSCs), as well as in auxiliary diagnosis and efficacy evaluation of orthopedic diseases. Lin et al. established a SERS platform for monitoring activity and quantitative analysis of alkaline phosphatase (ALP), an important diagnostic indicator for osteoporosis [28]. Sun et al. developed SERS nanosensors that could achieve in situ activity monitoring of alkaline phosphatase during osteogenic differentiation in BMSCs [29]. Cao et al. reported a SERS-based method for the long-term tracking of the dynamic expression of miR-144-3p in the osteogenic differentiation of BMSCs [30]. Jiang et al. explored a SERS-based lateral flow assay to detect the CTX I (an indicators of sensitivity in osteoporosis) with high sensitivity and specificity, and this method could not be interfered by serum matrix [31]. Over the last decade, our laboratory has developed a series of SERS substrates aimed at achieving highly sensitive detection of biomedical samples, including the analysis of biochemical changes in bone tissue during fracture healing [32], auxiliary diagnosis of knee osteoarthritis [33], and screening of cancer (e.g., liver cancer [34], breast cancer [35], nasopharyngeal carcinoma [36,37], prostate cancer [38], and bladder cancer and kidney cancer [39]). These previous studies demonstrated the feasibility of SERS technology in the auxiliary screening of PMOP. Among the various biological specimens commonly analyzed in clinical settings, serum is rich in biochemical components and easily obtainable, making it well-suited for screening PMOP. Therefore, in this study, serum samples were detected using high-sensitivity SERS technology to assist in PMOP screening. Compared with bone tissue biopsy or ex vivo bone sample testing, serum-based SERS only requires a trace amount of blood sample. This approach thus avoids invasive procedures. Moreover, when compared to blood/urine-based biomarker detection methods, SERS technology dispenses with the need for complex sample pretreatment, reducing the detection time to the minute level. Additionally, serum SERS detection can capture vibrational information from molecules such as proteins, nucleic acids, and lipids, which reflects the overall status of bone metabolism. As a result, it is more suitable for routine rapid clinical screening.

In this study, we applied SERS technology combined with multivariate statistical methods for the auxiliary screening of PMOP. Firstly, we constructed the PMOP rat model using ovariectomy (OVX), with a Sham group and an icariin (ICA) treatment group serving as controls. Subsequently, we prepared a highly homogeneous Au NPs monolayer film, and high-quality SERS signals were obtained from serum samples in the Sham, OVX, and ICA groups, with the purpose of exploring the specific changes in biochemical components associated with PMOP. Then, partial least squares (PLS) and support vector machine (SVM) were employed to classify the SERS signal acquired from the three serum groups, in order to investigate the feasibility of SERS technology in the screening of PMOP. This study will contribute to the development of an auxiliary screening method for PMOP based on SERS technology.

## 2. Materials and Methods

### 2.1. Rat Model and Treatment

All procedures were approved by the Bioethics Committee at Fujian University of Traditional Chinese Medicine (Approval Number: 3W2023122).

Ninety 3-month-old, specific pathogen-free (SPF) female Sprague-Dawley rats, weighing 300 ± 20 g, were purchased from Slac Laboratory Animal Co., Ltd. (Shanghai, China) (Production License No. SCXK 2022-0004). The rats were housed in the Experimental Animals Center of the Fujian University of Traditional Chinese Medicine. Feeding conditions were as follows: room temperature: 22–26 °C, relative humidity: 40–70%, and ad libitum access to food and water. In addition, ICA was purchased from Chengdu Must Bio-Technology Co., Ltd. (Chengdu, China) (Product ID: A0145). In total, 1 g ICA was added to 250 mL of 0.9% saline to prepare a 4 mg/mL suspension of ICA, which was stored at 4 °C for subsequent treatment.

After 1 week of acclimatization, 90 rats were randomly divided into the Sham, OVX, and ICA groups (n = 30 in each group). The rats in both the OVX and ICA groups underwent OVX surgery [40]. The experimental details for inducing the PMOP rat model by OVX surgery are shown in the Supplementary Materials. In the Sham group, without performing bilateral ovariectomy, only a small amount of adipose tissue around the ovaries was removed, and the rest of the operation was performed similarly to the OVX groups. After 1 week, all the rats in the Sham, OVX, and ICA groups received oral gavage dosing for 12 weeks. The treatments were as follows: in both the Sham and OVX groups, 0.9% saline was administered by gavage at 0.5 mL/100 g body weight q.d.; in the ICA group, a 4 mg/mL of ICA suspension was gavaged at 0.5 mL/100 g body weight q.d.

### 2.2. Collection of Serum and Bone Tissue Samples

After 12 weeks of treatment, the three groups of rats were fasted for 12 h. After achieving adequate anesthesia using isoflurane (2%), 2 mL of blood was collected from the abdominal aorta. After clotting for 30 min at room temperature, sera were centrifuged at 1000× *g* at 4 °C for 10 min, transferred to a clean centrifuge tube, and centrifuged again at 1000× *g* for 5 min to completely remove platelets and other precipitates. Sera were then collected and frozen in a −80 °C freezer for SERS detection. The third lumbar vertebra was obtained in each group, fixed in 4% paraformaldehyde, and used for hematoxylin–eosin (H&E) staining and Masson staining. The detailed experimental methods of H&E staining and Masson staining are presented in the Supplementary Materials.

### 2.3. Preparation and Characterization of Au NPs Monolayer Film

#### 2.3.1. Preparation of Au NPs Monolayer Film Based on the Marangoni Effect

**Figure 1:**
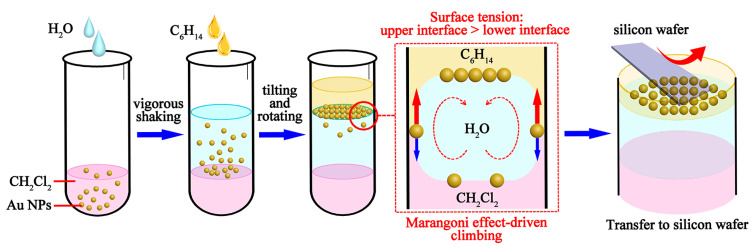
Schematic diagram of the Marangoni effect-driven self-assembly process of Au NPs at the oil/water/oil interface.

For serum SERS detection, Au NPs were used as the enhancement substrates for Raman scattering, and a highly homogeneous Au NPs monolayer film was prepared based on the Marangoni effect. Figure 1 illustrates a schematic diagram of the self-assembly process of Au NPs driven by the Marangoni effect at the oil/water/oil three-phase interface.

Firstly, according to the method reported by Frens [41], 1.5 mL of sodium citrate (mass fraction 1%) was added dropwise to 100 mL of boiling HAuCl_4_ solution (mass fraction: 0.01%). The solution was boiled and stirred for 15 min, followed by cooling to room temperature, and then the gold nanosol was obtained, exhibiting a wine-red color. Subsequently, Au NPs monolayer films were prepared using an oil/water/oil three-phase system based on the Marangoni effect [42,43,44]. A 1% polyvinyl pyrrolidone (PVP) solution was prepared by dissolving 1 g of PVP in 100 mL of anhydrous ethanol. Then, 1 mL of gold nanosol was centrifuged at 10,000 rpm, and the Au NPs were resuspended in 1 mL of 1% PVP solution, mixed evenly, and then allowed to stand for 10 min. This was followed by the centrifugation and washing of the Au NPs with anhydrous ethanol and resuspension in 1 mL of anhydrous ethanol. Thereafter, 100 μL of the Au NPs dispersed in anhydrous ethanol was evenly mixed with 1 mL of dichloromethane (CH_2_Cl_2_) in a centrifuge tube, followed by the addition of 1.8 mL of ultrapure water. As dichloromethane was immiscible with water, the Au NPs remained in the organic phase (CH_2_Cl_2_). After vigorous shaking for 30 s, a nondense membrane of Au NPs was formed at the CH_2_Cl_2_/H_2_O interface. Next, hexyl hydride (C_6_H_14_) was introduced as another type of oil phase into the CH_2_Cl_2_/H_2_O system. A total of 400 μL of C_6_H_14_ was added to the solution, and then the centrifuge tube was slightly tilted and slowly rotated. During this process, the surface tension of the upper oil/water interface (C_6_H_14_/H_2_O) was higher than that of the lower interface (CH_2_Cl_2_/H_2_O). Then, the Au NPs spontaneously entered the C_6_H_14_/H_2_O interface due to surface tension differences, resulting in the formation of a dense monolayer film. After the solution was left to stand and stratification occurred, the top layer of C_6_H_14_ was removed using a pipette, and then the Au NPs monolayer film could be easily transferred to silicon wafers. Finally, a silicon wafer was tilted and immersed in the solution, allowing the Au NPs film to attach to the silicon wafer. Then, the silicon wafer was removed from the solution, resulting in a silicon wafer with a Au NPs monolayer film on its surface. The silicon wafer was air-dried at room temperature and stored away from light for subsequent experiments.

#### 2.3.2. Characterization of Gold Nanosol and Au NPs Monolayer Film

The gold nanosol was characterized by absorption spectroscopy and dynamic light scattering (DLS). The absorption spectra of gold nanosol were recorded using a PerkinElmer Lambda 950 spectrophotometer (Waltham, MA, USA). The size and distribution of Au NPs were determined using a laser particle size analyzer (Malvern Zetasizer Nano ZS90, Malvern, UK). Furthermore, transmission electron microscopy (TEM) (JEM-2100Plus, Japan Electronics Co., Ltd., Tokyo, Japan) and scanning electron microscopy (SEM) (GeminiSEM 300, ZEISS, Oberkochen, Germany) were used, respectively, to characterize the morphology of Au NPs and the Au NPs monolayer film.

To test the SERS enhancement effect of the Au NPs monolayer film, 10 μL of 4-Mercaptobenzoic acid (4-MBA) solution (0.1 mM) was added dropwise to the silicon wafer loaded with Au NPs monolayer film and to a normal silicon wafer without Au NPs monolayer film. After drying at room temperature, the Raman signals of 4-MBA were detected under the same detection conditions. We further employed the Au NPs monolayer film to detect the SERS signals of 4-MBA solutions at various concentrations (10^−4^ M, 10^−5^ M, 10^−6^ M, 10^−7^ M, 10^−8^ M, and 10^−9^ M). Moreover, to verify the homogeneity and stability of SERS detection on Au NPs monolayer film, 100 points within the 4-MBA spotted region on the film were subjected to automatic SERS scanning detection. The automatic SERS scanning utilized a computer-controlled x-y moving platform in conjunction with a laser beam to scan the entire area. The scanning area was 20 μm × 20 μm, the laser beam spot size was adjusted with a pinhole to approximately 2 μm, and the scanning step size was set to 2 μm. The wavenumber range was 400–1800 cm^−1^. Additionally, to investigate the batch uniformity of the Au NPs monolayer film, five batches of the films were prepared and employed for SERS detection of 4-MBA (0.1 mM). From each batch, ten SERS spectra were recorded. The relative standard deviations (RSDs) of the main SERS peaks (1075 cm^−1^ and 1583 cm^−1^) were then calculated to assess batch-to-batch variability.

### 2.4. Serum SERS Measurements

For serum SERS detection, 10 μL of serum sample was added dropwise to the Au NPs monolayer film and allowed to air dry at room temperature. The SERS spectra of serum samples were detected using a Raman spectrometer (Renishaw, Gloucester, UK) with a 785 nm excitation wavelength. Spectra were collected in backscattering geometry via a microscope with a Leica 50× objective. Additionally, the detection of the Raman signal was performed using a Peltier-cooled CCD camera. For each measurement, the spectral integration time was set to 10 s, and the spectral detection range was 400 cm^−1^ to 1800 cm^−1^. The spectral resolution was 2 cm^−1^. The excitation light power was set to 0.1 mW.

In this study, there were 30 serum samples in each of the Sham group, OVX group, and ICA group. For each serum sample, 10 spectra were randomly detected and acquired within the serum droplet area. Then, the average spectrum, calculated by averaging the 10 collected spectra, represented the SERS signal of that serum sample. Finally, 30 averaged spectra (one per sample) were obtained from each group for subsequent analysis.

### 2.5. SERS Data Processing and Analysis

The raw signals contained background fluorescence and SERS signal. In order to extract the SERS signal, we employed the Vancouver Raman Algorithm to efficiently eliminate the background fluorescence [45]. After that, we performed normalization on the SERS spectra. Specifically, we first calculated the total intensity by integrating the whole SERS spectrum, taking the area under the spectrum as the integrated intensity value. Then, we divided the intensity at each spectral point by this integrated intensity value, thereby obtaining the normalized SERS spectrum. Finally, these normalized SERS spectra were subjected to spectral comparative analysis and multivariate statistical analysis using the PLS-SVM method.

PLS and SVM analyses were conducted using the MATLAB software package (Version R2016b). Firstly, we applied PLS to reduce the dimensionality of the spectral data and extract pertinent variables, which are referred to as PLS components (PCs). After that, the optimal number of PCs was determined by combining the mean squared error of prediction (MSEP) with the adjusted Wold’s R criterion [33,38]. Then, we fed the selected PCs into the SVM algorithm. In this study, the Gaussian Radial Basis Function (RBF) was used as the kernel function to classify different sample groups. Finally, we carried out sample discrimination analysis based on 10-fold cross-validation using the LIBSVM toolbox (version 3.23) [46]. Furthermore, the sensitivity, specificity, and accuracy in sample classification were calculated, and the diagnostic performance was evaluated by means of receiver operating characteristic (ROC) curves.

### 2.6. Statistical Analyses

Statistical analyses were conducted using the SPSS software (Version 19.0.0, IBM, Reston, VA, USA). Initially, normality tests (Shapiro–Wilk test) and homogeneity of variance test (Levene’s test) were performed on the data. If the data met the assumptions of normal distribution and homogeneity of variances, one-way ANOVA was used for multiple-group comparisons. Alternatively, if the data met the assumption of normal distribution but exhibited non-homogeneous variances, Welch’s ANOVA was employed. Furthermore, if the data did not meet the assumption of normal distribution, the Kruskal–Wallis test, a non-parametric test, was used for multiple-group comparisons. The results were presented using box plots, and a *p* value < 0.05 was considered statistically significant.

## 3. Results

### 3.1. Histomorphology Confirms the Successful Establishment of the PMOP Rat Model

**Figure 2:**
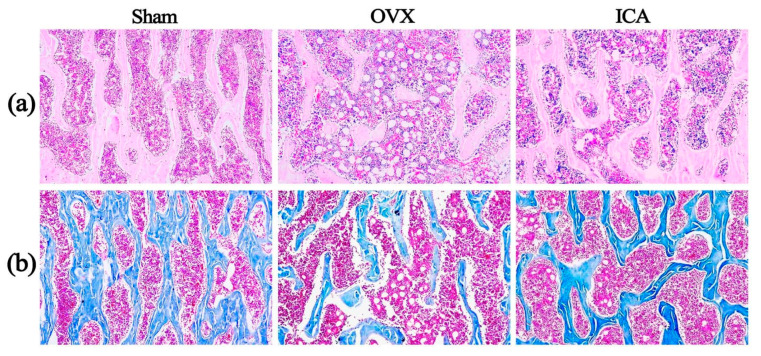
Hematoxylin–eosin (H&E) staining (**a**) and Masson staining (**b**) of the third lumbar vertebrae of rats from the Sham, OVX, and ICA groups, respectively (×100).

The results of HE staining and Masson staining in Figure 2 indicate that the Sham group presents an intact trabecular bone structure, characterized by uniformly stained bone collagen fibers, a high cellular density in the bone marrow, and few bone marrow fat vacuoles. Compared to the Sham group, the OVX group shows obvious trabecular bone fracture and thinning, uneven staining of collagen fibers, a decreased cellular density in the bone marrow, and a significant rise in bone marrow fat vacuoles. In contrast with the OVX group, the ICA treatment group demonstrates improved continuity and integrity of trabecular bone, relatively uniform staining of collagen fibers, an elevated cellular density in the bone marrow, and a decrease in bone marrow fat vacuoles. The above pathologic changes indicated that we successfully established the PMOP rat model by ovariectomy surgery. In addition, ICA treatment demonstrated a significant therapeutic effect on PMOP, although it did not provide full recovery.

### 3.2. Au NPs Monolayer Film Has Good SERS Enhancement Effect and Stability

**Figure 3:**
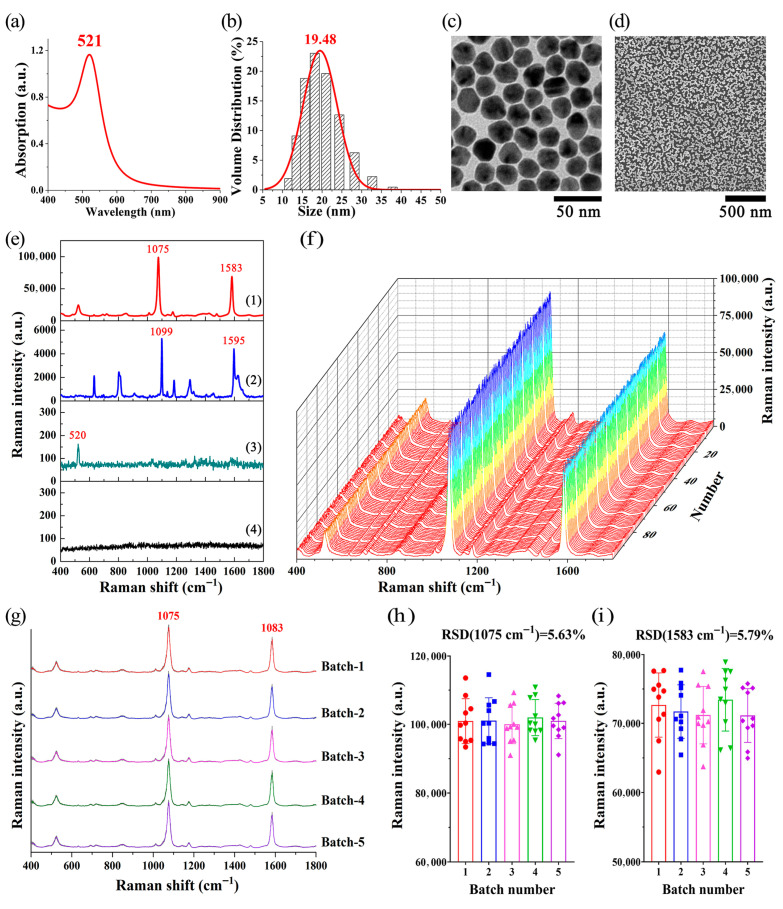
(**a**) Absorption spectra of Au NPs. (**b**) DLS results for Au NPs. (**c**) TEM image of Au NPs. (**d**) SEM image of Au NPs monolayer film. (**e**) SERS signal of 4-MBA on a Au NPs monolayer film-coated silicon wafer (spectral line 1); Raman signal of 4-MBA on a bare silicon wafer (spectral line 2); background signal of the Au NPs monolayer film (spectral line 3); and background signal of the Au NPs (spectral line 4). All of these spectra were obtained under the same detection conditions: 50× objective lens, excitation wavelength of 785 nm, laser power of 0.1 mW, and spectral integration time of 10 s. (**f**) SERS signals from 100 points within the 4-MBA spot region on the Au NPs monolayer film were detected using the SERS automatic scanning. (**g**) SERS spectra of 4-MBA obtained from five batches of Au NPs monolayer films. Each averaged spectrum is the mean of 10 spectra from the corresponding Au NPs monolayer film batch, with shaded areas showing the SD. (**h**,**i**) RSDs of the main SERS peak intensities for 4-MBA at 1075 cm^−1^ and 1583 cm^−1^ across the five batches.

Figure 3a shows that the maximum absorption wavelength of Au NPs is 521 nm. The DLS results (Figure 3b) indicate that the particle size distribution of Au NPs is about 19 nm. Figure 3c demonstrates that the Au NPs are spherical in shape and exhibit good dispersibility. The SEM image (Figure 3d) reveals that the Au NPs monolayer film on the silicon wafer has a monolayer distribution.

As shown in Figure 3e, spectral line 1 and spectral line 2 show the SERS signal of 4-MBA on a Au NPs monolayer film-coated silicon wafer (with SERS substrate) and the Raman signal of 4-MBA on a bare silicon wafer (without SERS substrate), respectively. Spectral lines 3 and 4 correspond to the background signals of the Au NPs monolayer film-coated silicon wafer and the Au NPs, respectively. All the spectra were obtained under identical detection conditions. In comparison to spectral line 1, the Raman scattering signal of 4-MBA on a regular silicon wafer, which lacks an SERS substrate, displayed significantly lower intensity (spectral line 2). In contrast, the Au NPs monolayer film exhibited significant enhancement of Raman scattering, allowing for the detection of Raman signal with an improved signal-to-noise ratio (spectral line 1), as evidenced by the robust signal intensity of the characteristic peaks of 4-MBA (1075 cm^−1^ and 1583 cm^−1^). Additionally, spectral line 4 indicates that Au NPs exhibit no significant background interference signals or Raman characteristic peaks within the range of 400–1800 cm^−1^. Meanwhile, the Raman peak at 520 cm^−1^ in spectral line 3 (Au NPs monolayer film) is the characteristic peak of silicon wafer (Si).

4-MBA is a commonly used molecular probe that is widely applied in Raman spectroscopy and SERS research. As indicated in Figure 3e, spectral line 2, the characteristic Raman peaks of 4-MBA are primarily determined by molecular vibration modes, such as aromatic ring stretching vibrations, carboxyl group (COOH) vibrations, and thiol group (SH) vibrations. Among them, the Raman spectral peak at 1099 cm^−1^ is assigned to the stretching vibration associated with the C-S bond; the peak at 1595 cm^−1^ is assigned to monosubstituted benzenes [47]. When 4-MBA molecules are adsorbed onto nanostructures, their SERS characteristic peaks often exhibit shifts. Research suggests that when 4-MBA adsorbs onto the surface of nanostructures, surface strain is generated, and the compressive or tensile strain induced by surface reconstruction can lead to shifts in the Raman peaks [48]. Other studies have also shown that charge transfer occurs at the interface between 4-MBA and nanomaterials, resulting in shifts in their SERS characteristic peaks [49]. The experimental observations in this study were consistent with previous reports, and the strong peaks at 1075 cm^–1^ and 1583 cm^–1^ can be assigned to aromatic ring vibrations.

To test the SERS enhancement effect of the Au NPs monolayer film, we further employed it to detect the SERS signals of 4-MBA solutions at various concentrations (10^−4^ M, 10^−5^ M, 10^−6^ M, 10^−7^ M, 10^−8^ M, and 10^−9^ M), as shown in Figure S2a–f. Figure S2g presents a plot of the intensity of the characteristic Raman peak of 4-MBA at 1075 cm^−1^ versus the logarithm of the 4-MBA concentration. Notably, when the concentration of 4-MBA was 10^−9^ M, the Au NPs monolayer film achieved an enhancement factor of approximately 10^6^, demonstrating its excellent SERS enhancement performance. The relevant experimental results have been included in the Supplementary Materials for further reference.

To further validate the stability of SERS assay of the Au NPs monolayer film, 10 μL of 4-MBA (0.1 mM) was added dropwise onto the Au NPs monolayer film and allowed to air dry at room temperature. SERS automatic scanning was used to detect SERS signals from 100 points in the area coated with 4-MBA. The results of SERS scanning are shown in Figure 3f, indicating robust SERS enhancement and good repeatability. We further calculated the RSD of intensities of 4-MBA characteristic Raman peak (1075 cm^−1^, assigned to the ring breathing of 4-MBA), which was 2.72%. Figure 3g presents the SERS signals acquired through the detection of 4-MBA (0.1 mM) using five batches of Au NPs monolayer films. Each averaged spectrum represents the mean of 10 spectra measured from the corresponding batch of Au NPs monolayer films, with shaded areas indicating the standard deviation (SD). Moreover, the RSDs of the main SERS peak intensities have been calculated. As shown in Figure 3h,i, for the 50 SERS spectra measured from five batches of Au NPs monolayer films, the RSDs of the intensities of the characteristic SERS peaks of 4-MBA at 1075 cm^−1^ and 1583 cm^−1^ remain below 5.8%. Additionally, the original SERS data obtained from the five batches of Au NPs monolayer films are provided in the Supporting Information (Figure S3). These findings suggest that the Au NPs monolayer film exhibits a robust SERS enhancement effect, along with good uniformity and stability, making it suitable for application in biological sample assays.

### 3.3. Differences in Serum SERS Spectra Between Sham, OVX, and ICA Treatment Groups

**Figure 4:**
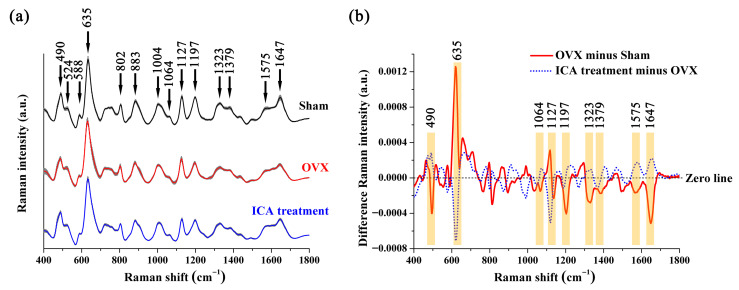
Comparison of serum SERS spectra collected from the Sham, OVX, and ICA treatment groups: (**a**) The average SERS spectra for each of these three sample groups, with the shaded area indicating the standard deviation of the SERS signal. (**b**) Spectral differences between the OVX group and Sham group, and those between the ICA treatment group and OVX group. Significant variations in the SERS spectra are highlighted in orange.

Figure 4a shows the average SERS signal and standard deviation of the rat serum in the Sham, OVX, and ICA groups. Some SERS peaks were observed in all three sample groups, such as 490, 524, 588, 635, 802, 883, 1004, 1064, 1127, 1197, 1323, 1379, 1575, and 1647 cm^−1^. Table 1 displays the primary SERS peak positions and their tentative assignments based on some studies [39,50,51,52]. Figure 4b depicts the spectral variations between the OVX and Sham groups, and between the ICA treatment and the OVX groups. In Figure 4b, the orange regions highlight the notable distinctions in SERS bands among different sample groups, including alterations in both Raman shifts and band intensity. These variations were primarily associated with lipids, amino acids, carbohydrates, and collagen.

**Figure 5:**
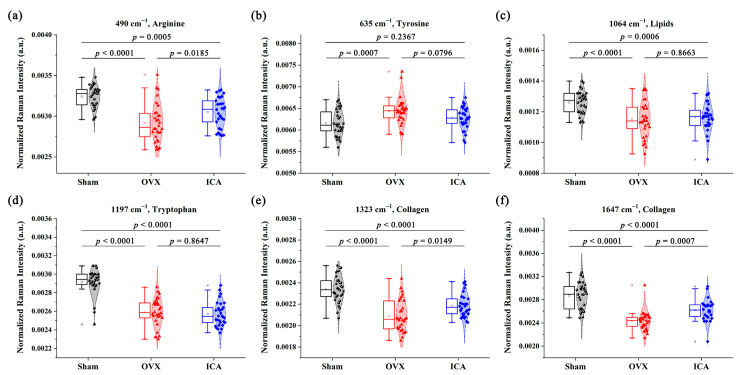
Significantly different SERS peaks were compared by box plots between the Sham, OVX, and ICA groups. (**a**) Arginine; (**b**) Tyrosine; (**c**) Lipids; (**d**) Tryptophan; (**e**) Collagen; (**f**) Collagen.

Using box plots (as shown in Figure 5), we conducted a further quantitative analysis of the SERS peaks that displayed notable differences. Peak at 635 cm^−1^ (tyrosine) had higher intensity in the OVX group than that in the Sham group. On the contrary, the intensities of SERS peaks at 490 cm^−1^ (Arginine), 1064 cm^−1^ (Lipids), 1379 cm^−1^ (Lipids), 1197 cm^−1^ (Tryptophan), 1323 cm^−1^ (Collagen), and 1647 cm^−1^ (Collagen) were lower in the OVX group than in the Sham group. Further comparison of trends in biochemical components among the three groups showed that ICA treatment ameliorated some biochemical changes caused by OVX, although they were not fully restored to Sham levels. Taken together, differences in the SERS signals were evident among the three sample groups, indicating that these spectral characteristics could be utilized to analyze the subtle variations in the biochemical compositions of serum and assist in the auxiliary screening of PMOP.

### 3.4. PMOP Screening Based on PLS-SVM Statistical Analysis

**Figure 6:**
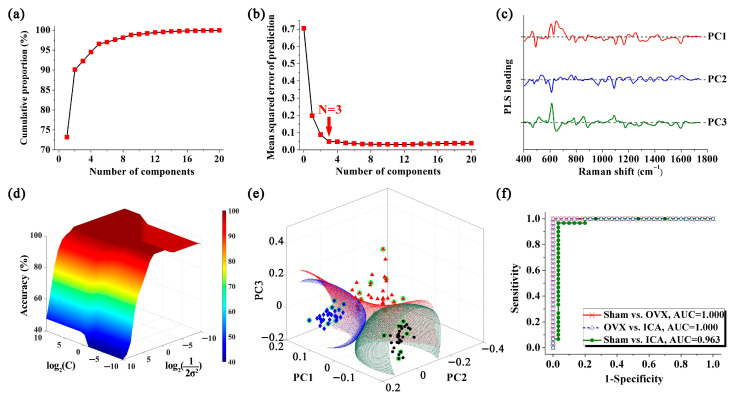
PMOP discrimination based on PLS-SVM analysis: (**a**) The cumulative proportion of PLS components. (**b**) The mean squared error of prediction (MSEP) of PLS components. (**c**) The loadings of PC1, PC2 and PC3, respectively. (**d**) 3D plot of the discrimination accuracy from the SVM approach by varying the Gaussian radial widths *σ* and penalty factor *C*. (**e**) SVM discrimination results (black spheres: Sham group; red triangles: OVX group; blue diamonds: ICA treatment group; green circles: support vectors). (**f**) ROC curves for categorical outcomes of different groups.

Given that the SERS spectra of different sample groups exhibited significant similarities, it was inadequate to distinguish them merely by directly observing the spectral lines. Consequently, we utilized the PLS-SVM algorithm to extract spectral information and classify the samples, thereby facilitating the screening of PMOP. The main goal of PLS in this study was to downscale the dimensionality of SERS spectra and obtain a series of PLS components (PCs). Figure 6a displays the cumulative proportion of the PCs derived from the PLS algorithm. However, building a diagnostic model with an excessive number of PCs may lead to over-fitting. Studies have indicated that the mean squared error of prediction (MSEP) is a statistically reliable approach for selecting the optimal number of PCs in PLS [53]. Figure 6b illustrates that the MSEP curve of PLS exhibited two distinct phases. In the initial phase, MSEP decreased rapidly, while in the subsequent phase, the rate of decrease significantly slowed down. Following this, the adjusted Wold’s R criterion was employed to ascertain the most suitable number of PCs for constructing the diagnostic discriminant model [53,54]. According to the adjusted Wold’s R criterion, new PCs will not be incorporated into the model unless they lead to a significant improvement in predictions. According to MSEP_N=4_ < 5%, the cutoff point of the MSEP curve was determined to be located at PC3, resulting in the utilization of only PC1, PC2, and PC3 for modeling the identification of PMOP. The loadings for the first three PCs are displayed in Figure 6c.

To classify the samples, an SVM analysis was performed using the Gaussian radial basis function (RBF) kernel. In order to construct the optimal classifier, two crucial parameters of the SVM, namely the Gaussian radial widths *σ* and penalty factor *C*, were fine-tuned through a grid search approach [55]. The 3D plot shown in Figure 6d depicts the discrimination accuracy for various values of *σ* and *C*. Figure 6e illustrates the discrimination of the three sample sets through hyperplanes produced by SVM analysis. In this figure, samples from the Sham, OVX, and ICA treatment groups were represented by black spheres, red triangles, and blue diamonds, respectively, while the support vectors were marked with green circles. The SVM algorithm generated hyperplanes that were capable of accurately identifying and classifying serum samples from different groups. Using this method, we conducted discriminant analysis for the comparisons of Sham vs. OVX, OVX vs. ICA, and Sham vs. ICA, and achieved favorable binary classification results in all cases. Additionally, receiver operating characteristic (ROC) curves were employed to validate the discrimination of OVX. As depicted in Figure 6f, the areas under the ROC curve for the comparisons of Sham vs. OVX, OVX vs. ICA, and Sham vs. ICA were 1.000, 1.000, and 0.963, respectively.

**Figure 7:**
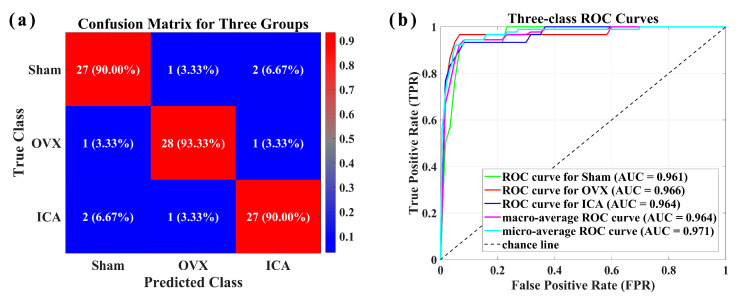
(**a**) Confusion matrix for the three sample groups. (**b**) ROC curve and AUC value for the classification of the three sample groups.

We further performed multi-class classification, utilizing the PLS-SVM algorithm to classify serum SERS data from the Sham, OVX, and ICA groups. The classification results are shown in Figure 7. The Confusion matrix (Figure 7a) and the receiver operating characteristic (ROC) curve (Figure 7b) demonstrate that using the PLS-SVM algorithm, OVX can be discriminated with high sensitivity (93.33%) and high specificity (96.67%) when classifying three sample groups simultaneously. The three-class classification achieved an accuracy of 91.11% (82/90). Table 2 provided a summary of the discrimination results, which encompassed sensitivity, specificity, and accuracy. The aforementioned results indicated that the combination of SERS with PLS-SVM multivariate analysis possessed significant potential for serving as an auxiliary screening tool for PMOP.

## 4. Discussion

PMOP is a metabolic bone disease caused by estrogen deficiency and is characterized by a generalized decrease in bone density and microstructural degeneration of bone tissue. An imbalance between bone formation and resorption is a major contributor to PMOP development, and various factors such as sex hormones and inflammatory responses are also involved [56,57]. With the aging of the population, the incidence of PMOP is expected to rise, representing a significant burden on both patients and the healthcare system. Early screening and intervention for PMOP can help prevent the occurrence of fragility fractures.

We used a combination of the oil/water/oil three-phase system with the Marangoni effect to prepare the Au NPs monolayer film. In this three-phase system, the Marangoni force drove the transport and compression of Au NPs, which originated from the surface tension difference between the two oil/water interfaces. When the surface tension of the upper interface (C_6_H_14_/H_2_O) is higher than that of the lower interface (CH_2_Cl_2_/H_2_O), the combined force pulls the Au NPs up to the upper oil/water interface (as shown in Figure 1). Additionally, some studies demonstrated that the Marangoni force could overcome the electrostatic repulsion between the Au NPs, resulting in a high-density monolayer film [44]. In this study, based on the oil/water/oil three-phase system, we achieved the preparation of highly reproducible, large-area Au NPs monolayer films with excellent consistency across different preparation batches. The obtained Au NPs monolayer films could serve as uniform and highly active substrates, enhancing the sensitivity of SERS detection. Therefore, the rapid preparation of Au NPs monolayer films using the oil/water/oil three-phase system contributed to promoting high-throughput and low-cost screening and diagnosis for PMOP.

In this study, SERS spectroscopy revealed a decrease in serum arginine levels in the OVX rat model, which rebounded following ICA intervention (as depicted in Figure 5a). There is a correlation between L-arginine and osteoporosis, particularly PMOP. A study using mildly aged and OVX mouse models found that L-arginine can mitigate bone loss, promote osteoblast differentiation and angiogenesis, while inhibiting BMSC differentiation into adipocytes [58]. In the OVX mice, L-arginine supplementation significantly improved bone mineral density and trabecular bone structure, indicating its therapeutic potential for PMOP. The mechanism involves L-arginine alleviating oxidative stress and protecting osteoblast and endothelial cell functions through PINK1/Parkin and Bnip3-mediated mitophagy pathways. Additionally, studies show that L-arginine inhibits TNF-α-induced osteoclastogenesis by reprogramming metabolism, thereby reducing inflammatory bone destruction. It promotes a metabolic shift in osteoclasts from glycolysis to oxidative phosphorylation, increasing ATP and purine metabolite levels (e.g., hypoxanthine and inosine) while blocking osteoclast differentiation [59]. Furthermore, metabolites related to L-arginine metabolism are altered in the serum of rheumatoid arthritis patients, suggesting their potential regulatory role in human osteoporosis.

Conversely, the SERS peak at 635 cm^−1^, attributed to tyrosine, increased in the OVX group and significantly decreased after ICA treatment. Research indicates that tyrosine can activate anabolic pathways related to bone remodeling under normal physiological conditions, contributing to the maintenance of bone mass [60]. In postmenopausal women, due to the decline in estrogen levels, tyrosine metabolic disturbances are closely associated with the development of PMOP. Ling et al. utilized targeted metabolomics to compare fecal samples from osteoporosis patients and healthy controls, finding that the tyrosine content in feces from the lumbar spine osteoporosis group was significantly higher than that in the control group (*p* < 0.05) [61]. This suggests that tyrosine metabolism may be altered in the context of bone loss. The alteration in tyrosine metabolism can be attributed to metabolic imbalances caused by hormonal fluctuations, which may interfere with amino acid metabolism (including tyrosine), leading to decreased bone mineral density and an increased risk of fractures. However, another study focusing on the elderly population found that alterations in serum tyrosine levels are associated with the risk of osteoporosis. Among elderly men, higher serum tyrosine levels are correlated with a reduced risk of osteoporosis (a 24% risk reduction) [62]. Therefore, further research is needed to investigate the relationships between biomarkers in different clinical samples and the pathogenesis of osteoporosis, particularly changes in characteristic metabolites of osteoporosis.

The level of serum tryptophan (detected at 1197 cm^−1^) was also notably decreased in the OVX group, yet ICA treatment could not elevate it back to the level observed in the Sham group. There is a “protective” association between tryptophan and bone mineral density, supported by relevant evidence-based research. Population-based cohort studies demonstrate an inverse relationship between tryptophan intake and the risk of postmenopausal osteoporosis. Su et al. collected data on bone mineral density (BMD) and tryptophan intake from 12,003 participants. They analyzed these data to evaluate the impact of tryptophan intake on the risk of low bone mass (LBMD) in middle-aged and elderly individuals. The study found that tryptophan intake was significantly lower in participants with LBMD compared to those with normal bone mass, and a negative correlation existed between tryptophan intake and the risk of LBMD [63]. Through plasma metabolomics analysis of 135 participants, Yang et al. further confirmed a positive correlation between circulating tryptophan levels and bone density. As plasma tryptophan levels increased, the risk of osteoporosis decreased [64]. Additionally, studies have shown that tryptophan can regulate osteoblast activity, mesenchymal stem cell differentiation, and osteoclastogenesis through the serotonin and melatonin pathways, thereby contributing to the maintenance of bone mass after menopause [65].

Collagen is one of the main components of bone, and a decrease in its content may lead to the destruction of the bone microstructure, thereby affecting bone density and bone strength, and increasing the risk of fractures. The results of Masson staining of bone tissue sections (Figure 2b) revealed that the Sham group exhibited an intact trabecular bone structure with uniformly stained bone collagen fibers. In comparison to the Sham group, the OVX group showed obvious trabecular bone fractures and uneven staining of collagen fibers. We further employed the ELISA method to quantitatively analyze the levels of the bone formation marker Procollagen I N-terminal peptide (PINP) and the bone resorption marker β-C-terminal telopeptide of type I collagen (β-CTX) in the serum samples of rats from each group. The detection procedures and operational steps for each marker were carried out strictly in accordance with the instructions provided in the reagent kits. The ELISA results (Figure S1 in the Supplementary Materials) revealed that compared with the Sham group, the OVX group exhibited a significant decrease in the serum level of the bone formation marker PINP and a significant increase in the serum level of the bone resorption marker β-CTX. These differences were statistically significant (*p* < 0.05). These findings were consistent with a previous research report [66]. Furthermore, in this study, compared to the Sham group, the OVX group exhibited diminished SERS peak intensities at 1323 cm^−1^ and 1647 cm^−1^, which are attributed to the CH_3_CH_2_ wagging mode and the amide I (α-helix) of collagen, respectively. On the contrary, a marked elevation in SERS intensity at 1647 cm^−1^ was observed in the ICA group, indicating that ICA intervention had the potential to enhance collagen synthesis. Some reports also indicated that collagen levels in the OVX group were markedly reduced compared to the Sham group, and these alterations could be mitigated by ICA treatment [67,68]. The aforementioned results demonstrated that high-sensitivity SERS was capable of identifying subtle alterations in the biochemical components of serum during the progression of PMOP.

To validate the feasibility of using SERS for PMOP screening, diagnostic discrimination was performed on SERS signals using PLS-SVM analysis. PLS was first utilized to reduce the dimensionality of high-dimensional spectral data into several PCs, which retained information on sample characteristics. In the field of spectral data dimensionality reduction, PLS and Principal Component Analysis (PCA) are two commonly used methods. PCA is an unsupervised statistical method that extracts the most important features by maximizing data variance during data dimensionality reduction. Conversely, PLS is a supervised modeling approach that aims to identify a set of components that can maximize the explained variance of the response variable while minimizing the variance of the predictor variables. Compared to PCA, PLS incorporates the influence of the response variable during the dimensionality reduction process. This allows PLS to better explain the direction of the predictor variables and accurately predict the dependent variable, providing an advantage over PCA in terms of model interpretability and predictive performance. However, using all principal components (PCs) generated by the PLS algorithm to construct a classification model may lead to over-fitting. Therefore, in this study, the MSEP curve was used to determine the optimal number of PCs [69]. Finally, PC1, PC2, and PC3 were input into the SVM algorithm for sample classification.

Figure 6e demonstrates that the SVM algorithm was capable of effectively categorizing serum samples from the Sham, OVX, and ICA treatment groups. Compared to traditional linear classification approaches, such as linear discriminant analysis (LDA), SVM is capable of handling complex nonlinear classification problems. SVM introduces kernel functions, which can map samples from the original feature space to a higher-dimensional feature space, making the data linearly separable in this new space. Then, the SVM algorithm aims to find an optimal hyperplane that maximizes the distance between the two classes of samples closest to the hyperplane on either side, thereby providing good generalization capability and robustness for sample classification. Briefly, the identification of OVX achieves 100% in sensitivity, specificity, and diagnostic accuracy, highlighting the significant potential of combining SERS detection with PLS-SVM-based spectral data analysis for the auxiliary screening of PMOP.

## 5. Conclusions

In this study, we explored the feasibility of using the SERS assay as an auxiliary diagnostic tool for PMOP. We combined the oil/water/oil three-phase system with the Marangoni effect to prepare Au NPs monolayer films to obtain high-quality serum SERS spectra from the Sham, OVX, and ICA treatment groups of the rat model. The Au NPs monolayer films exhibited robust SERS enhancement as well as good uniformity and stability. SERS spectra effectively revealed subtle differences in serum biochemical compositions across various sample groups, demonstrating their potential for assessing PMOP-related expression characteristics. Through spectral difference analysis, biochemical alterations linked to PMOP were identified, such as the upregulation of tyrosine expression and the downregulation of arginine, tryptophan, some lipids, and collagen. By combining SERS spectra with PLS-SVM analysis, we achieved remarkable sensitivity, specificity, and an accuracy rate of 100% in screening OVX samples. This study confirmed the feasibility of using SERS technology combined with multivariate statistical analysis methods in the screening of PMOP.

Next, we plan to utilize the SERS technique to analyze serum samples collected from normal subjects, subjects with reduced bone mass, and subjects with osteoporosis. We will further improve the SERS substrate by incorporating an internal standard and applying it to serum SERS detection and analysis. We will utilize Raman probe molecules with characteristic peaks located in the silent region (1800–2800 cm^−1^) as the internal standard. Since their signals fall into a different spectral range from those of most biochemical molecules, this approach can significantly avoid signal interference while providing a reference peak with stable intensity, thereby enhancing the accuracy of quantitative detection. We believe that our series of research will contribute to the auxiliary screening of PMOP.

## Figures and Tables

**Table 1:** SERS Peak positions and tentative assignments.

Peak Positions (cm^−1^)	Tentative Assignments
490	Arginine: S-S stretching vibration
524	Amino acid cysteine: S-S stretching mode
588	Ascorbic acid, Amide VI
635	Tyrosine: C-S stretching vibration
802	Uracil-based ring breathing mode
883	CH_2_, protein assignment
1004	Phenylalanine: Ring breathing
1064	Lipids: Skeletal C-C stretch
1127	D-mannose: C-N stretching vibration
1197	Tryptophan: ring vibration
1323	Collagen: CH_3_CH_2_ wagging mode
1379	Lipids: CH_3_ symmetric
1575	DNA/RNA bases: Ring breathing modes
1647	Collagen, Amide I: α-Helix

**Table 2:** Discrimination results of PLS-SVM analysis.

Sample Groups	Sensitivity	Specificity	Accuracy
Sham vs. OVX	100%	100%	100%
OVX vs. ICA	100%	100%	100%
Sham vs. ICA	96.67%	96.67%	96.67%
Sham vs. OVX vs. ICA	93.33%	96.67%	91.11%

## Data Availability

The dataset and code used in this research can be obtained by contacting the authors.

## Supplementary Materials

The following supporting information can be downloaded at https://www.mdpi.com/article/10.3390/bios15090568/s1, including the establishment of the postmenopausal osteoporosis rat model and the process of H&E staining and Masson staining. Figure S1: The changes in the levels of bone formation marker Procollagen I N-terminal peptide (PINP) and bone resorption marker β-C-terminal telopeptide of type I collagen (β-CTX) in rat serum; Figure S2: Concentration-dependent SERS spectra of 4-MBA at different concentrations on the Au NPs monolayer films; Figure S3: SERS signals of 4-MBA detected using five batches of Au NPs monolayer films.
