# Supplementary material for: A Highly Sensitive SERS Technique Based on Au NPs Monolayer Film Combined with Multivariate Statistical Algorithms for Auxiliary Screening of Postmenopausal Osteoporosis
Source: Biosensors (Basel). 2025 Aug 30;15(9):568. doi: 10.3390/bios15090568 (PMC12467781; doi:10.3390/bios15090568)
Supplement: Supplementary file 1 [file biosensors-15-00568-s001.zip › biosensors-3802974-supplementary.docx]

**S**upplementary

A Highly Sensitive SERS Technique Based on Au NP
Monolayer Film Combined with Multivariate Statistical
Algorithms for Auxiliary Screening of Postmenopausal
Osteoporosis

Yun Yu ^1,2,3,^*, Jinlian Hu ^1^, Qidan Shen ^1^, Huifeng Xu ^1,2,3^, Shanshan Wang ^1,2,3^, Xiaoning Wang ^1,2,3^,
Yuhuan Zhong ^1,2,3^, Tingting He ^1,2,3^, Hao Huang ^1,2,3^, Quanxing Hong ^1,2,3^, Erdan Huang ^4^ and Xihai Li ^2,^*

^1^ Academy of Integrative Medicine, Fujian University of Traditional Chinese Medicine, Fuzhou 350122, China; jlianei99@163.com (J.H.); shenqidan1234567@126.com (Q.S.); xuhf@fjtcm.edu.cn (H.X.); 2020044@fjtcm.edu.cn (S.W.); 2007020@fjtcm.edu.cn (X.W.); yuhuan_zhong@126.com (Y.Z.); c2019078@fjtcm.edu.cn (T.H.); 1990021@fjtcm.edu.cn (H.H.); 2003011@fjtcm.edu.cn (Q.H.)

^2^ College of Integrative Medicine, Fujian University of Traditional Chinese Medicine, Fuzhou 350122, China

^3^ Fujian Key Laboratory of Integrative Medicine on Geriatrics, Fuzhou 350122, China

^4^ Fuzhou Second General Hospital, Fuzhou 350007, China; 18150067268@163.com

***** Correspondence: yuyunsatan@163.com (Y.Y.); 2010031@fjtcm.edu.cn (X.L.)

1. Establishment of postmenopausal osteoporosis rat model

After 1 week of acclimatization, 90 rats were randomly divided into the sham, OVX, and ICA treatment groups, with 30 rats in each group.

Rats in both the OVX and ICA treatment groups underwent ovariectomy (OVX) [1]. To accomplish this, the rats were anesthetized with isoflurane (2%), and then disinfected. Subsequently, an incision of approximately 1.0 cm was made at the intersection of the anterior edge of the pubic bone and the midline of the bilateral lower abdomen of the rats, and layers were dissected to expose the abdominal cavity. Rat ovaries were visible adjacent to the uterus, near the end of the fallopian tube. After partial dissection of the fat mass attached to the periphery of the ovary to fully expose it, the fallopian tube at the lower end of the ovary was clamped with hemostatic forceps and ligated with sutures. Then, both ovaries were completely removed. After observing no active bleeding, the uterus was gently retracted back into the abdominal cavity, layers were sutured, and blood around the incision was wiped away with gauze. Finally, penicillin was injected intramuscularly for 3 consecutive days (200,000 units/mouse q.d.) to prevent infections.

In the sham group, only a small amount of adipose tissue surrounding the ovaries was removed, without performing bilateral ovariectomy. The remainder of the operation was performed similarly to that in the OVX and ICA treatment groups.

2. Process of H&E staining and Masson staining

(1) Fixation: Samples were fixed in 4% paraformaldehyde for 48 h at room temperature and rinsed 3 times with distilled water.

(2) Decalcification: Decalcification was performed with 10% ethylenediamine tetraacetic acid at room temperature away from light, and the solution was changed every 2 days for 8 weeks until the texture resembled soft tissue.

(3) Paraffin embedding: Paraffin embedding was performed through alcohol gradient dehydration, xylene clearing, paraffin immersion, and embedding. Then, the samples were frozen at −20°C for 30 minutes.

(4) Tissue sectioning: Samples were sectioned at a thickness of 4 μm and spread on distilled water at 38°C to allow the sections to expand fully. Then, sections were collected using adhesive glass slides and baked at 60°C for 3 h.

(5) The sections were dewaxed and hydrated, followed by H&E and Masson staining.

(6) H&E staining: Paraffin sections were dewaxed and hydrated, stained with hematoxylin for 30 s, and rinsed with distilled water for 10 min; then, they were differentiated for 2-3 s using differentiation solution, immersed in distilled water for 5 min, stained with eosin for 2-3 s, and rinsed with distilled water for 10 min. The sections were then dried and sealed with neutral resin to observe the morphology and structure of the third lumbar vertebra under the microscope.

(7) Masson staining: The dewaxed and hydrated sections were stained with Bouin’s solution at 60℃ for 2 h and then rinsed with distilled water until the yellow color disappeared from the sections. Sections were stained with celestine blue staining solution for 3 min and rinsed with distilled water for 5 min. Then, the sections were stained with Mayer’s hematoxylin staining solution for 30 s and rinsed with distilled water for 5 min. Sections were differentiated with a differentiation solution for 1 s and rinsed with distilled water for 5 min. Then, the sections were stained with Ponceau S for 2 min, rinsed with distilled water for 5 min, and treated with a phosphomolybdic acid solution for 30 min until the collagen fibers stained with red were differentiated into colorless or light red color. Thereafter, sections were stained with aniline blue for 3 min and rinsed with distilled water for 5 min. The sections were dried and mounted with neutral resin. Then the morphological changes of the lumbar vertebrae in each group were observed under the microscope.

**Reference**

1. Yousefzadeh, N.; Kashfi, K.; Jeddi, S.;Ghasemi, A. Ovariectomized rat model of osteoporosis: a practical guide. *Excli journal*, **2020**, 19, 89-107. https://doi.org/10.17179/excli2019-1990


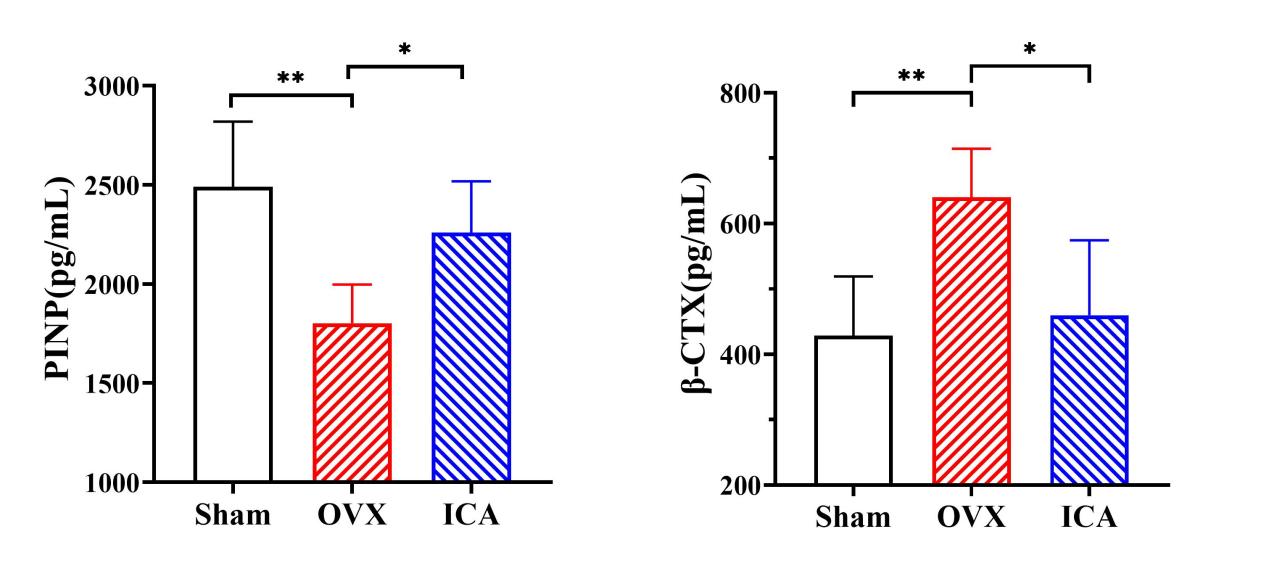


**Figure S1.** The changes in the levels of bone formation marker Procollagen I N-terminal peptide (PINP) and bone resorption marker β-C-terminal telopeptide of type I collagen (β-CTX) in rat serum. ^*^*P* < 0.05, ^**^*P* < 0.01.


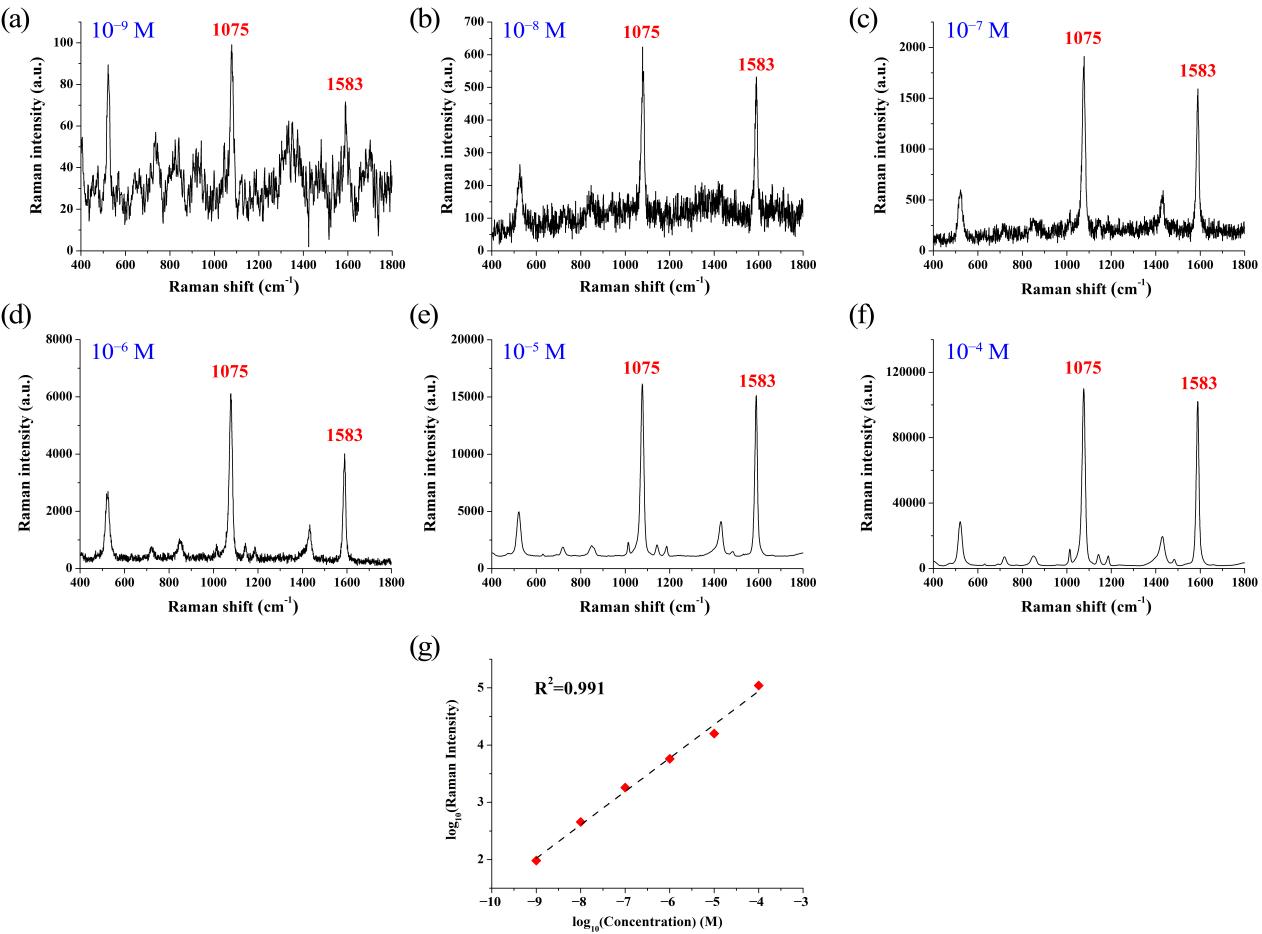


**Figure S2.** Concentration-dependent SERS spectra of 4-MBA at different concentrations on the Au NP monolayer films. (a) 10^−4^ M; (b) 10^−5^ M; (c) 10^−6^ M; (d) 10^−7^ M; (e) 10^−8^ M; (f) 10^−9^ M. (g) Logarithmic plot of the 1075 cm⁻¹ peak intensity versus 4-MBA concentration.


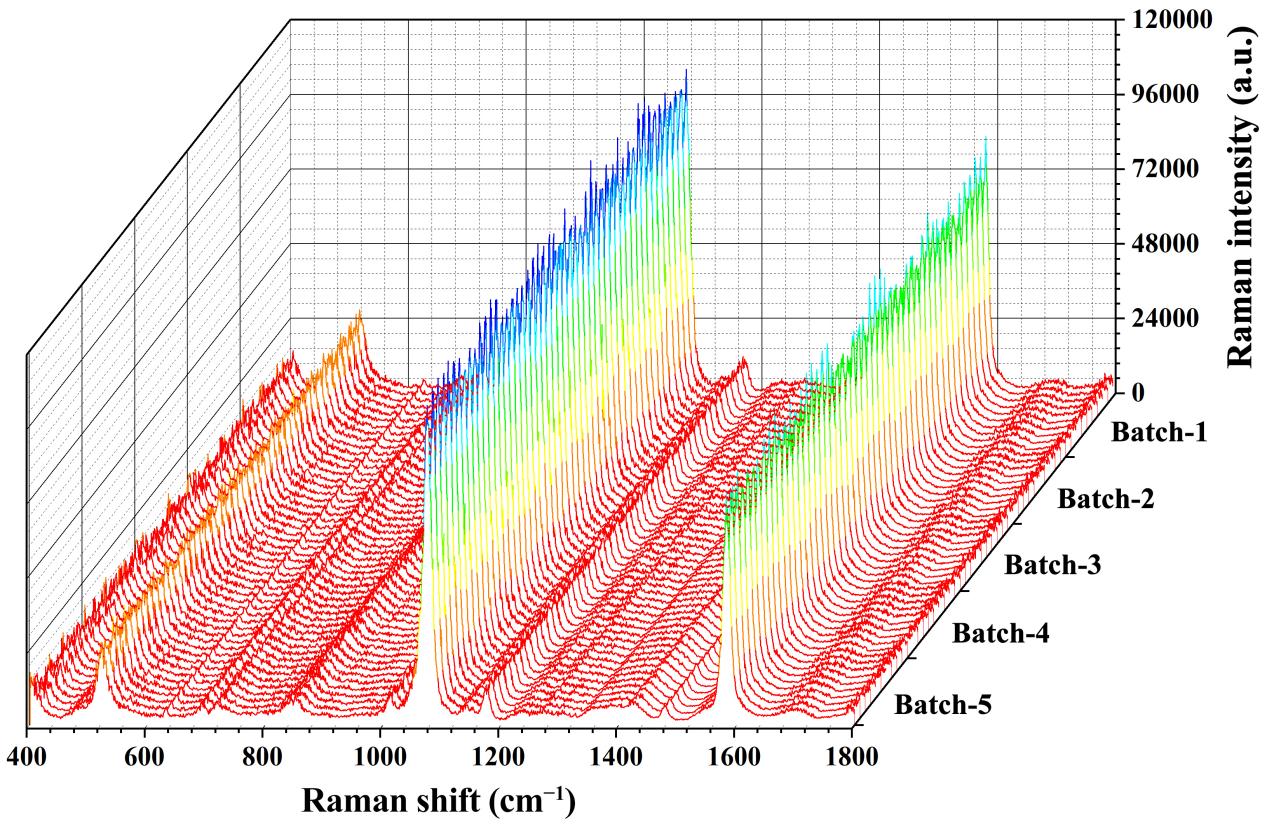


**Figure S3.** SERS signals of 4-MBA detected using five batches of Au NP monolayer films. Ten spectra per batch were acquired for analysis.

**Disclaimer/Publisher’s Note:** The statements, opinions and data contained in all publications are solely those of the individual author(s) and contributor(s) and not of MDPI and/or the editor(s). MDPI and/or the editor(s) disclaim responsibility for any injury to people or property resulting from any ideas, methods, instructions or products referred to in the content.
